# Kampo Formula-Pattern Models: The Development of 13 New Clinically Useful Standard Abdominal Pattern Models in the Fukushin Simulator

**DOI:** 10.3389/fphar.2022.688074

**Published:** 2022-04-29

**Authors:** Shuji Yakubo, Masaki Baba, Hiroshi Odaguchi, Akino Wakasugi, Mariko Sekine, Toshihiko Hanawa, Tadamichi Mitsuma, Takao Namiki, Makoto Arai, Shin-Ichi Muramatsu, Yutaka Shimada, Naotoshi Shibahara

**Affiliations:** ^1^ Department of Clinical Kampo Medicine, Meiji Pharmaceutical University, Tokyo, Japan; ^2^ Oriental Medicine Research Center, Kitasato University, Tokyo, Japan; ^3^ Department of Kampo Medicine, Aizu Medical Center, Fukushima Medical University, Fukushima, Japan; ^4^ Department of Japanese-Oriental (Kampo) Medicine, Graduate School of Medicine, Chiba University, Chiba, Japan; ^5^ Department of Kampo Medicine, Tokai University School of Medicine, Kanagawa, Japan; ^6^ Division of Oriental Medicine, Jichi Medical University, Tochigi, Japan; ^7^ Department of Japanese Oriental Medicine, Graduate School of Medicine and Pharmaceutical Sciences, University of Toyama, Toyama, Japan; ^8^ Division of Kampo Diagnostics, Institute of Natural Medicine, University of Toyama, Toyama, Japan

**Keywords:** Kampo (traditional Japanese herbal medicine), simulator, abdominal diagnosis, medical education, training, standardisation, abdominal palpation

## Abstract

**Aim:** In Kampo medicine, there exists an important system of diagnosis called Fukushin, or abdominal diagnosis or palpation. By applying pressure to the abdomen of the patient, the physician can gain important information on the patient’s physical state and use those indications to choose a suitable Kampo formulation. We have previously developed a Fukushin simulator, a teaching tool that reproduces the important abdominal patterns that doctors will encounter in clinical practice and that has received favourable feedback for students and practitioners. In order to make diagnosis and prescription easier, it is desirable to have matched formula–pattern pairings. The present study aims to develop such pairings.

**Methods:** With the previously developed models as a foundation, in the present study the production team (two members) used materials such as urethane foam and silicone rubber to build an additional 13 standard abdominal pattern models matched to Kampo herbal formulas commonly used by practitioners in Japan. Subsequently, the evaluation team (the remaining 10 authors) investigated the viability of these models.

**Results:** The evaluation team determined that abdominal pattern models matched to the following typical Kampo formulas were created successfully: Dai-saiko-To (大柴胡湯), Dai-joki-To (大承気湯), Shigyaku-San (四逆散), Saiko-ka-ryukotsu-borei-To (柴胡加竜骨牡蛎湯), Keishi-bukuryo-Gan (桂枝茯苓丸), Hachimi-jio-Gan (八味地黄丸), Hange-shashin-To (半夏瀉心湯), Sho-saiko-To (小柴胡湯), Hochu-ekki-To (補中益気湯), Sho-kenchu-To (小建中湯), Toki-shakuyaku-San (当帰芍薬散), Ninjin-To (人参湯), and Dai-kenchu-To (大建中湯).

**Conclusion:** We suggest that these new formula-pattern models can make an important contribution to the standardization of abdominal diagnosis and prescription and to Kampo education.

## Introduction

**FIGURE 1:**
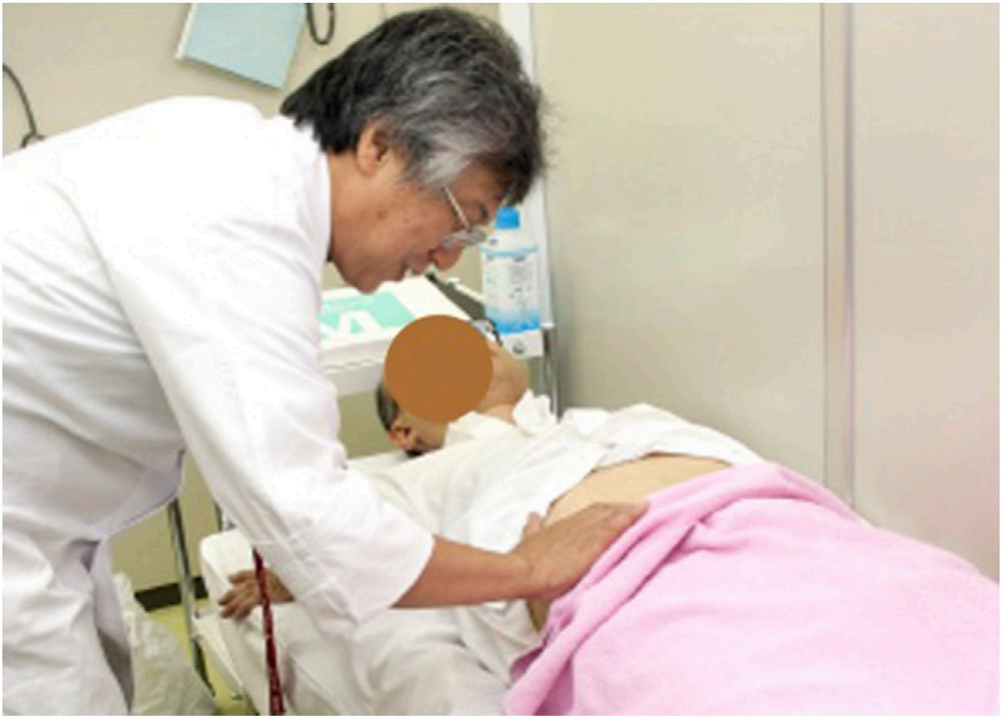
The Fukushin method. In Fukushin, the physician applies gentle pressure to the abdomen of the patient with knees stretched, guaging the resistance presented by the patient both overall and at specific sites, and using the information to derive an abdominal pattern. Written informed consent was obtained from the individual(s) for the publication of any identifiable image or data included this article.

A diagnostic method favored in Kampo medicine, one that originated in ancient China but has been developed independently in Japan, is called Fukushin, or abdominal palpation (Figure 1). It is used in clinical practice with all kinds of conditions. The physician applies gentle pressure to the abdomen of the patient, gauging the resistance presented by the patient both overall and at specific sites, and using that information to derive an abdominal pattern, which the physician uses to decide on a suitable Kampo formulation (Terasawa, 1993; The Japan Society for Oriental Medicine, 2005; Ushiroyama, 2005; Protnikoff and WatanabeYashiro, 2008; Arai et al., 2017).

There have been attempts to evaluate abdominal palpation through various medical tests, and some researchers have even attempted to develop equipment especially for that purpose (Tosa et al., 1982; Arichi et al., 1983; Shintani et al., 1989; Yasaka, 1994; Miyamoto and Okita, 2005; Nishida et al., 2012), but these attempts have generally foundered.

**FIGURE 2:**
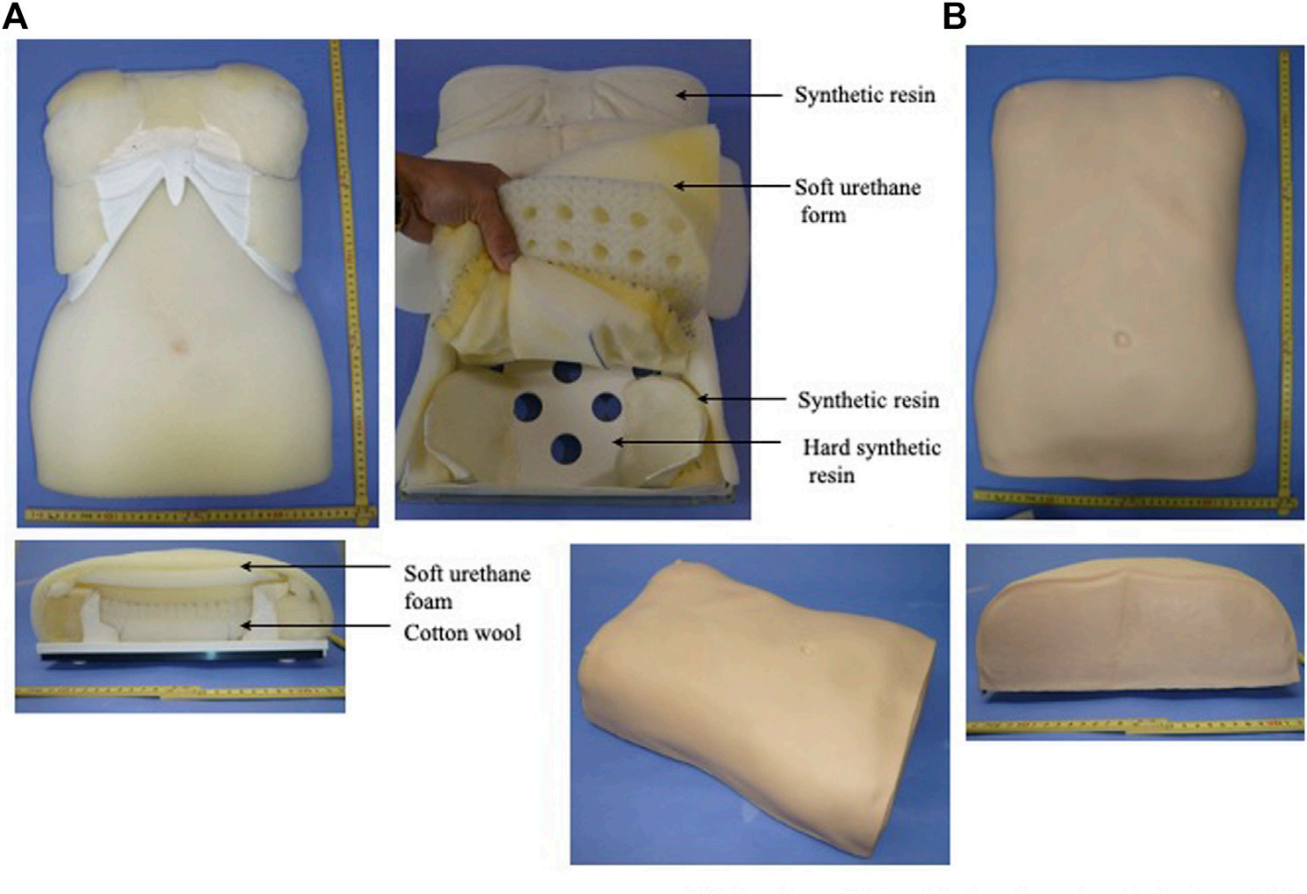
Fukushin simulator female body model. **(A)** Interior of Fukushin simulator female body model. **(B)** Exterior of Fukushin simulator female body model.

Because it is not possible to communicate with precision the sensations in an experienced practitioner’s hands when performing abdominal palpation, it has been established that education in abdominal palpation is challenging. To help that cause, as previously reported, we have developed a Fukushin simulator consisting of a set of models that reproduce the most important abdominal patterns that students will encounter in their future clinical practice (Figure 2) (Yakubo et al., 2008).

**FIGURE 3:**
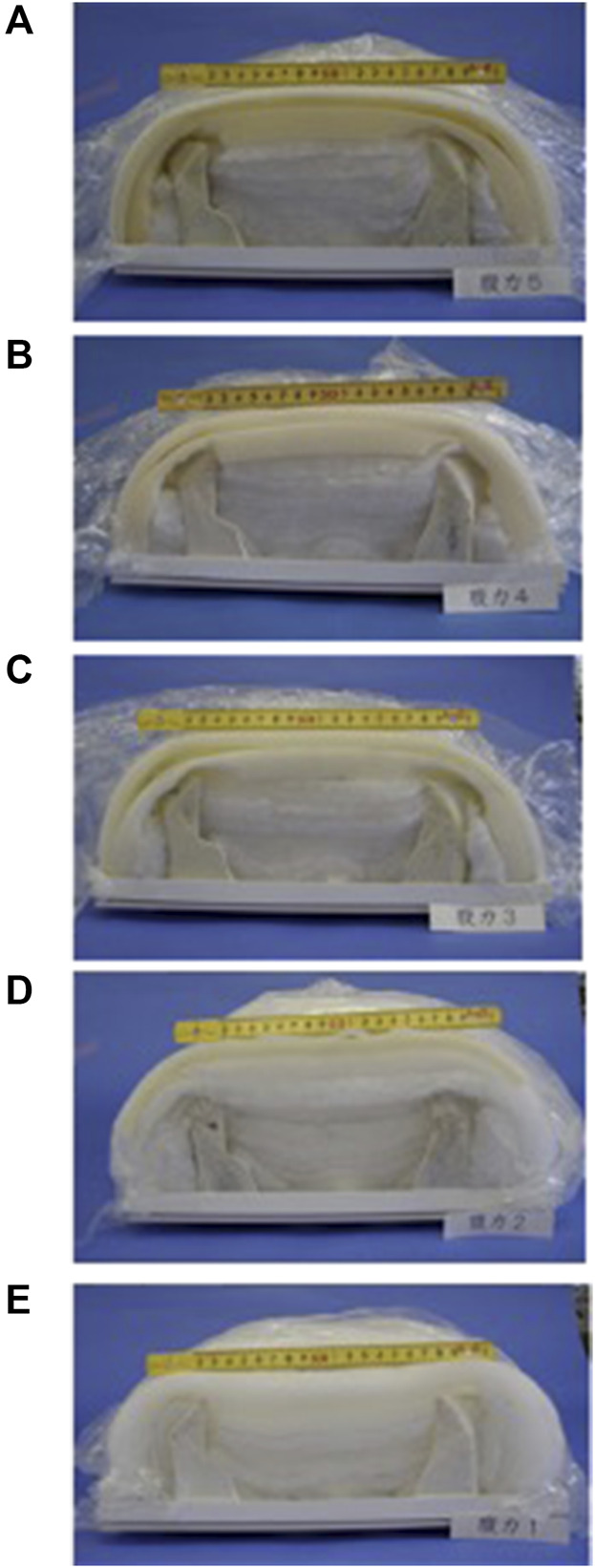
Interior of abdominal strength models. **(A)** An obvious excess abdominal pattern model. **(B)** A slight excess abdominal pattern model. **(C)** An intermediate excess abdominal pattern model. **(D)** A slight deficiency abdominal pattern model. **(E)** An obvious deficiency abdominal pattern model.

The simulator includes five models representing overall abdominal strength: the obvious excess model, the slight excess model, the intermediate model, the slight deficiency model, and the obvious deficiency model (Table 1) (Figure 3) (Yakubo et al., 2013).

**TABLE 1:** Abdominal strength pattern models.

Obvious excess abdominal strength pattern model: strongest resistance of abdominal wall and obvious abdominal distension
Slight excess abdominal strength pattern model: somewhat strong resistance of abdominal wall
Intermediate abdominal strength pattern model: neither strong nor weak resistance of abdominal wall
Slight deficiency abdominal strength pattern model: somewhat weak resistance of abdominal wall
Obvious deficiency abdominal strength pattern model: weakest resistance of abdominal wall and retraction of abdomen

Based on the intermediate abdominal strength model, we also developed the Fullness in the chest and hypochondrium model, the Stuffiness and rigidity below the heart model, the Rectus muscle tension model, the Lower abdominal fullness model, and the Lower abdominal numbness model (Yakubo et al., 2015). To the deficiency models, we also added models incorporating an Abdominal fluid congestion system and an Abdominal palpitation system (Table 2) (Yakubo et al., 2014a).

**TABLE 2:** Specific abdominal pattern models.

Stuffiness and rigidity below the heart model: Increased resistance in the epigastric region
Fullness in the chest and hypochondrium model: Increased resistance on both sides of the hypochondriac region
Rectus muscle tension model: Increased resistance in the area corresponding to the abdominal rectus muscle
Lower abdominal fullness model: A horseshoe-shaped area of markedly increased resistance in the lower abdomen
Lower abdominal numbness mode: Diminished resistance in the center of the lower abdomen
Abdominal fluid congestion model: A splashing sound is heard on tapping the abdomen
Oketsu tenderness model: Increased resistance and tenderness in the lower abdomen and peri-umbilical region
Abdominal palpitation model: A pulsating sensation can be felt in the abdomen

**FIGURE 4:**
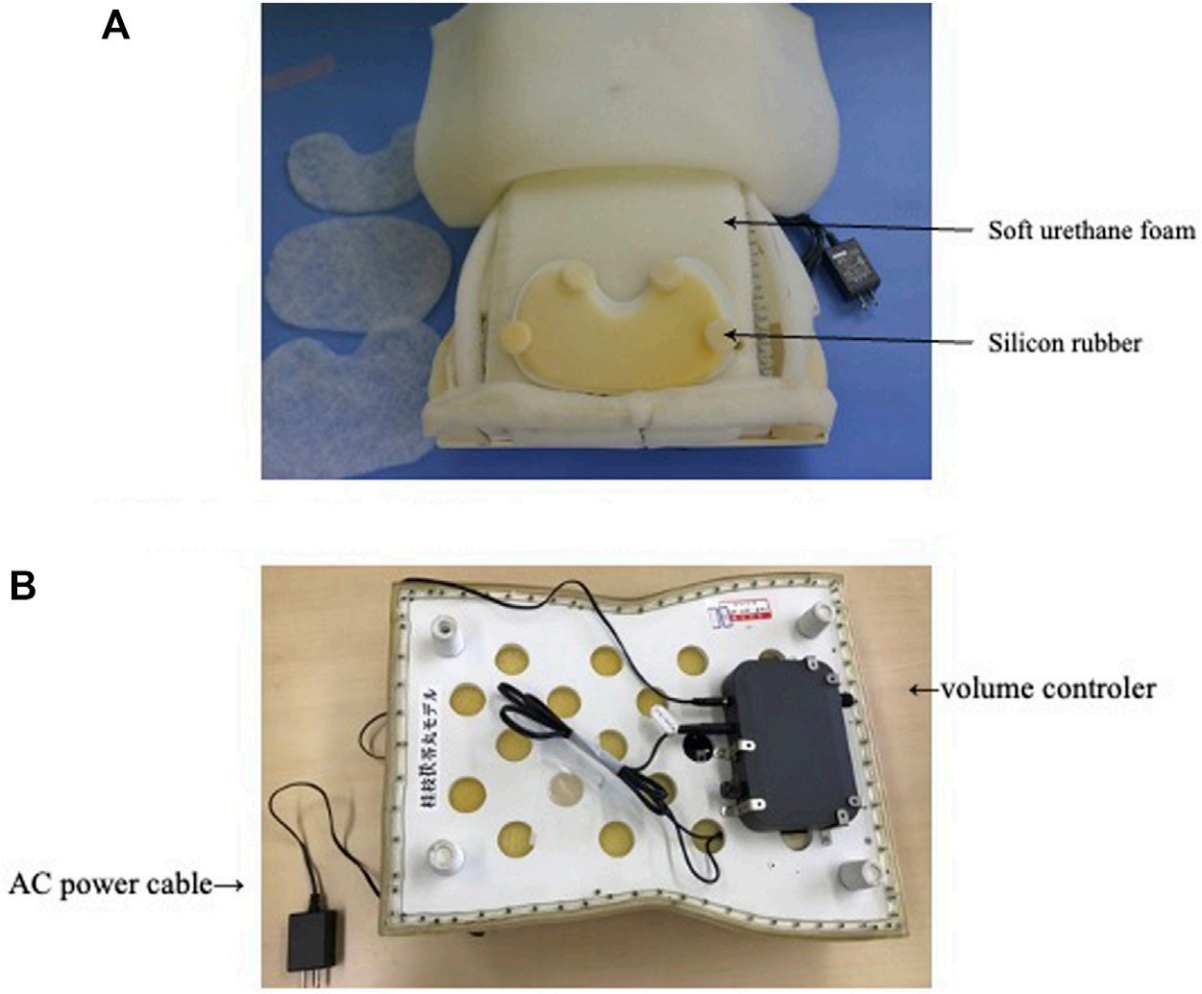
Keishinbukuryo-Gan pattern model. **(A)** The interior of the Keishi-bukuryo-Gan pattern model. **(B)** The reverse of the Keishi-bukuryo-Gan pattern model.

Because the abdominal patterns uncovered through abdominal palpation leads to prescriptions of standardized Kampo formulas, we have added new models, which we might call formula-pattern models, targeted at popular formulas, such as the Keishi-bukuryo-Gan (桂枝茯苓丸) and Toki-shakuyaku-San (当帰芍薬散) formula-pattern models, both corresponding to formulas frequently prescribed to women. In this spirit, a new model representing a woman’s body incorporating an Oketsu tenderness system was developed (Figure 4) (Baba et al., 2019).

The present paper describes recent development work conducted on the foundation of the work described above. We have developed a new version of the simulator that consists of 13 abdominal models, including the recently developed Keishi-bukryo-Gan and Toki-shakuyaku-San formula-pattern models, all of female bodies, representing standard abdominal patterns corresponding to well-known formulas (Table 3).

**TABLE 3:** 13 Important formula abdominal pattern models.

1. Obvious excess abdominal strength models
(a) Dai-saiko-To formula-pattern model
(b) Dai-joki-To formula-pattern model
2. Slight excess abdominal strength models
(a) Shigyaku-San formula-pattern model
(b) Saiko-ka-ryukotsu-borei-To formula-pattern model
(c) Keishi-bukuryo-Gan formula-pattern model
3. Intermediate abdominal strength models
(a) Hachimi-jio-Gan formula-pattern model
(b) Hange-shashin-To formula-pattern model
(c) Sho-saiko-To formula-pattern model
4. Slight deficiency abdominal strength models
(a) Hochu-ekki-To formula-pattern model
(b) Sho-kenchu-To formula-pattern model
(c) Toki-shakuyaku-San formula-pattern model
5. Obvious deficiency abdominal strength models
(a) Ninjin-To formula-pattern model
(b) Dai-kenchu-To formula-pattern model

## Materials and Methods

As shown in Table 3, Yakubo and Baba, the production team, created 13 formula-pattern models. These are all female models corresponding to 13 standard formulas, based on the female models previously mentioned.

**FIGURE 5:**
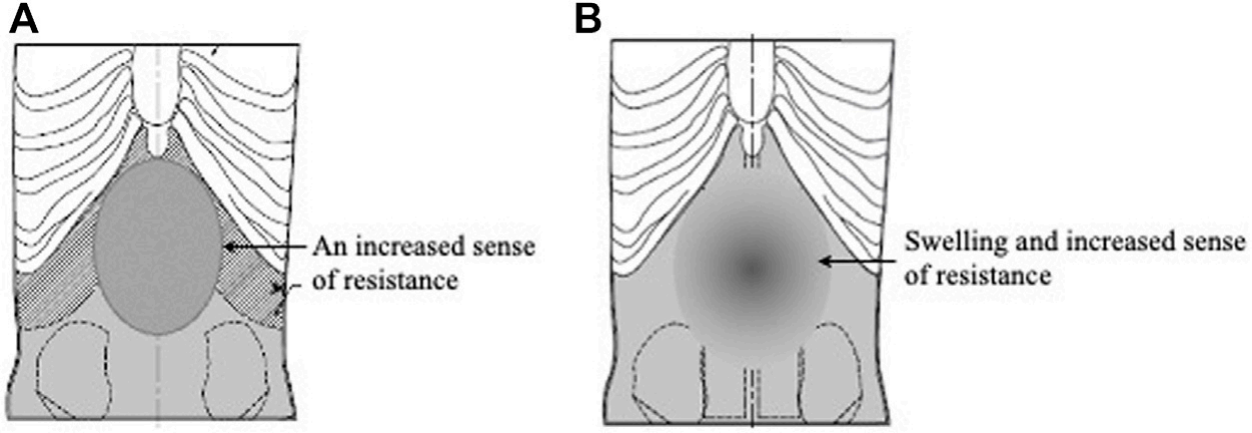
Obvious excess abdominal strength models. **(A)** Dai-saiko-To formula-pattern model. **(B)** Dai-joki-To pattern model.

They first created two models representing obvious excess. For the Dai-saiko-To (大柴胡湯) formula-pattern model, they inserted urethane foam and silicone rubber widely in the hypochondriac and epigastric regions (Figure 5A). For the Dai-joki-To (大承気湯) pattern model, they further increased the amount of urethane in the abdomen centered around the umbilical region (Figure 5B).

**FIGURE 6:**
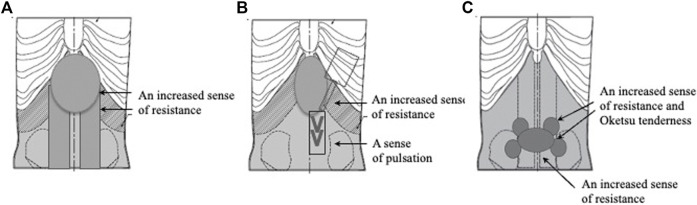
Slight excess abdominal strength models. **(A)** Shigyaku-San pattern model. **(B)** Saiko-ka-ryukotsu-borei-To pattern model. **(C)** Keishi-bukuryo-Gan pattern model.

The next three models all represent patterns of slight excess. For the Shigyaku-San (四逆散) formula-pattern model, they inserted urethane foam and silicone rubber in the lower hypochondrial and epigastric regions and to represent the abdominal rectus muscle (Figure 6A). For the Saiko-ka-ryukotsu-borei-To (柴胡加竜骨牡蛎湯) formula-pattern model, they also inserted urethane foam and silicone rubber in the lower hypochondrial and epigastric regions and added the Abdominal palpitation model previously mentioned (Figure 6B). For the Keishi-bukuryo-Gan formula-pattern model, they added urethane foam parts to the lower abdomen and also the previously mentioned Oketsu tenderness system (Figure 6C).

**FIGURE 7:**
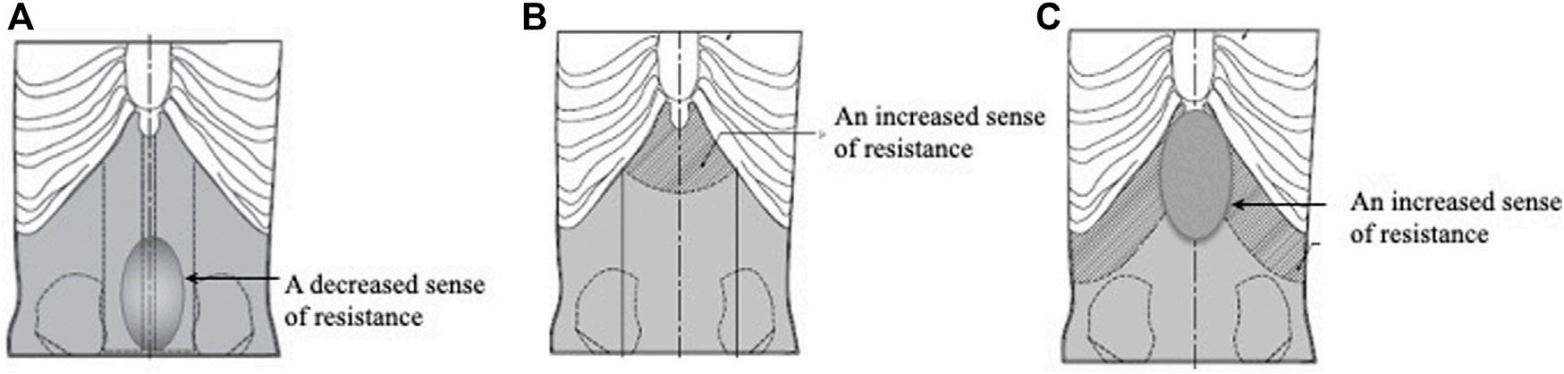
Intermediate abdominal strength models. **(A)** Hachimi-jio-Gan pattern model. **(B)** Hange-shashin-To pattern model. **(C)** Sho-saiko-To pattern model.

**FIGURE 8:**
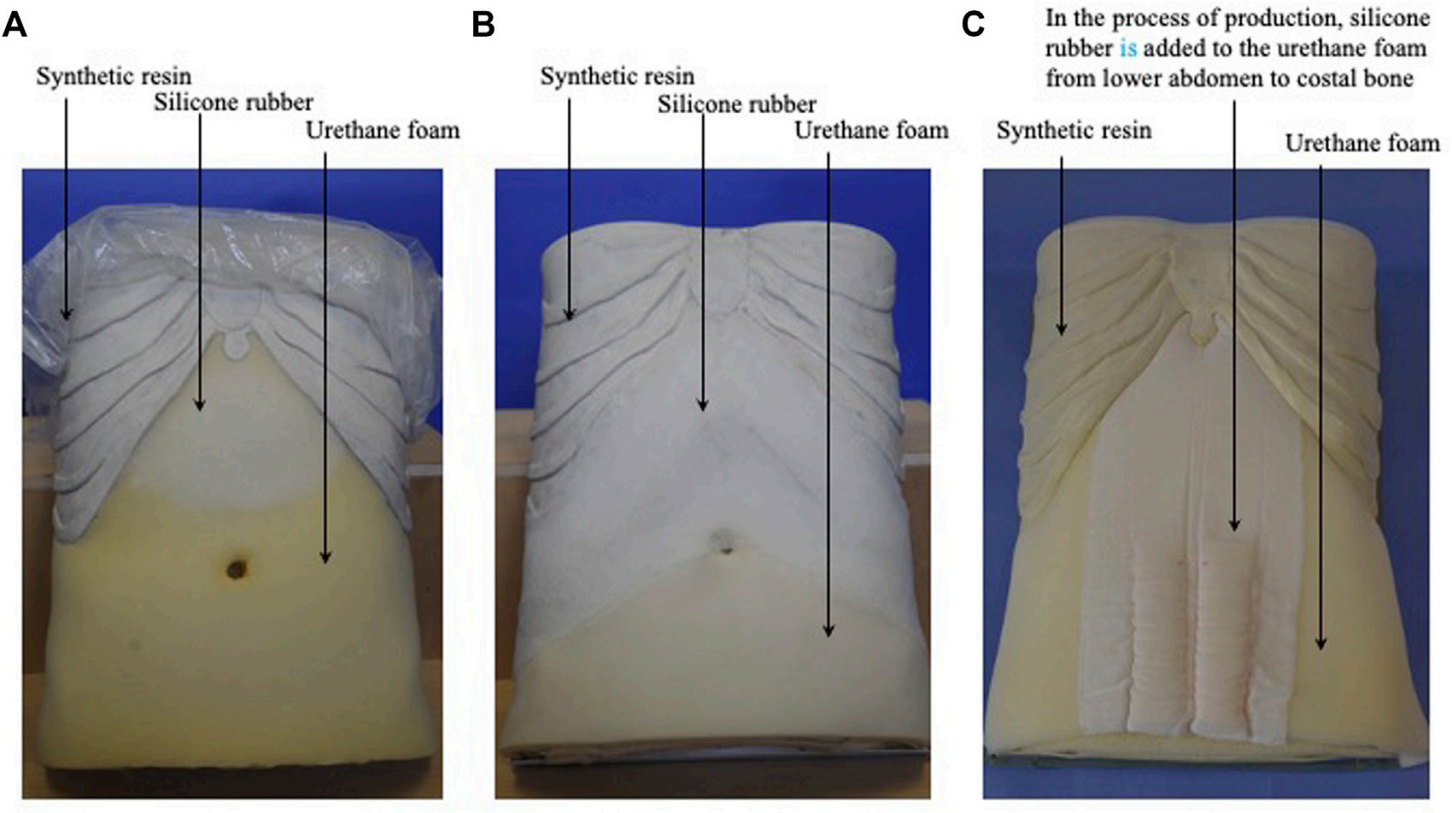
The interior of the Fukushin simulators. **(A)** The interior of the Hange-shashin-To formula-pattern model. **(B)** The interior of the Hochu-ekki-To formula-pattern model. **(A)** The interior of the Sho-kenchu-To formula-pattern model in the process production.

The next three models represent intermediate abdominal strength patterns. For the Hachimi-jio-Gan (八味地黄丸) formula-pattern model, they decreased the amount of urethane foam in the midline of the lower abdomen (Figure 7A). For the Hange-shashin-To (半夏瀉心湯) formula-pattern model, they added silicone rubber to the urethane foam in the epigastric region (Figures 7B, 8A). For the Sho-saiko-To (小柴胡湯) formula-pattern model, they added silicone rubber to the urethane foam in the lower hypochondrium and the epigastrium (Figure 7C).

**FIGURE 9:**
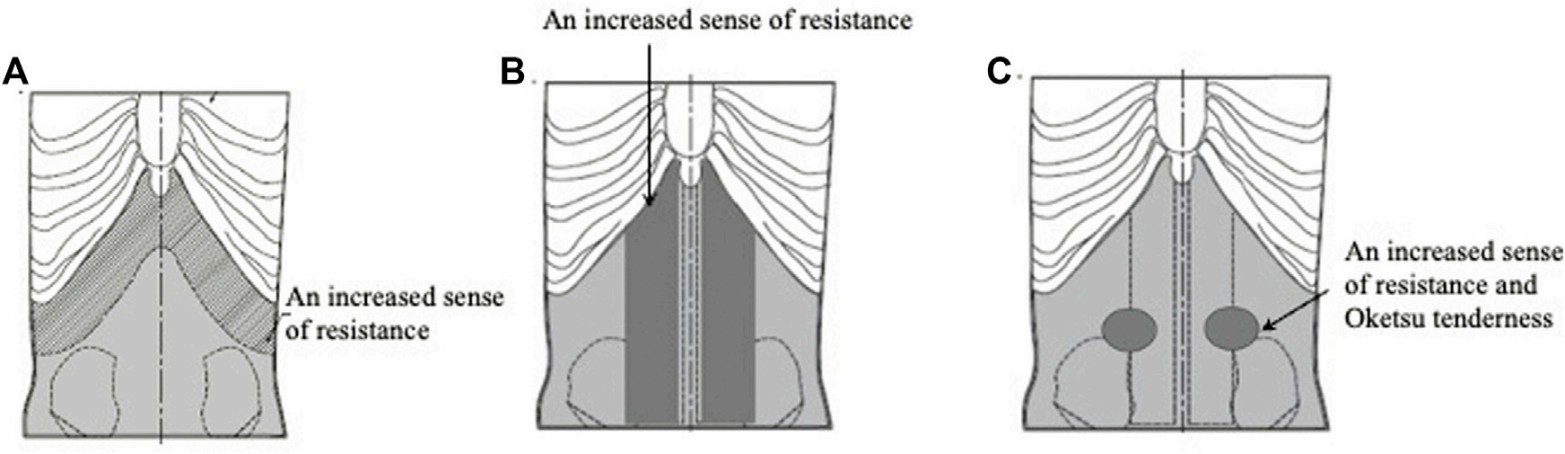
Slight deficiency abdominal strength models. **(A)** Hochu-ekki-To pattern model. **(B)** Sho-kenchu-To pattern model. **(C)** Toki-shakuyaku-San pattern model.

The next three models represent slight deficiency. For the Hochu-ekki-To (補中益気湯) formula-pattern model, they added silicone rubber to the urethane rubber in the lower hypochondriac region (Figures 8B, 9A). For the Sho-kenchu-To (小建中湯) formula-pattern model, they added silicone rubber to the urethane foam in the area representing the abdominal rectus muscle (Figures 8C, 9B. For the Toki-shakuyaku-San formula-pattern model, they added an Oketsu tenderness system as with the Keishi-bukuryo-Gan formula-pattern model (Figure 9C).

**FIGURE 10:**
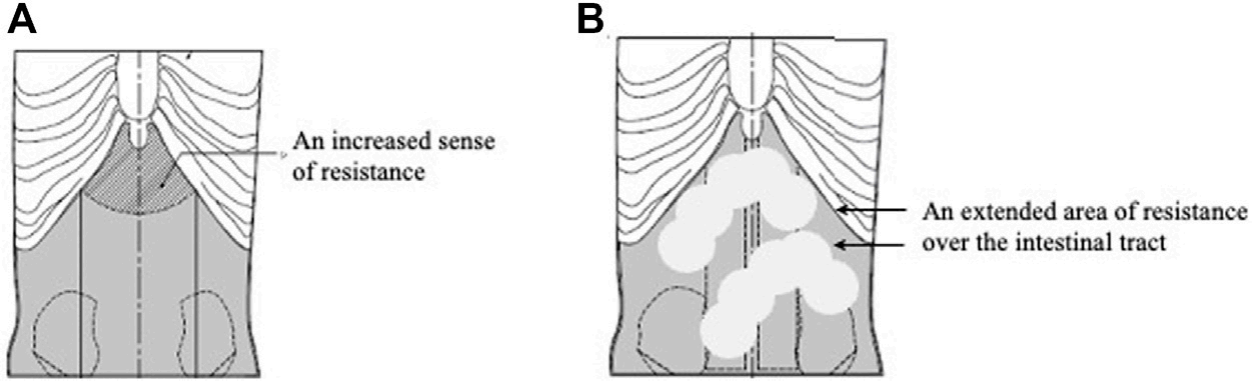
Obvious deficiency abdominal strength models. **(A)** Ninjin-To pattern model. **(B)** Dai-kenchu-To pattern model.

Finally, they created two obvious deficiency models. For the Ninjin-To (人参湯) formula-pattern model (Figure 10A), they added silicone rubber to the urethane foam in the epigastric region. For the Dai-kenchu-To (大建中湯) formula-pattern model, they packed cotton into an elastic tubular bag 5 cm in diameter to realize the expanded area of tension in the intestinal tract area characteristic of this abdominal pattern (Figure 10B).

Subsequently, the 10 members of the evaluation team investigated the new models by palpating them, judging whether the models were representations of the 13 standard Kampo formula abdominal patterns.

## Results

In the present research, the production team worked on 13 female abdominal models corresponding to 13 clinically significant Kampo formulations, and then the evaluation team investigated the models by palpating them.

With the two obvious excess models, the evaluators found that the Dai-saiko-To formula-pattern model featured increased resistance over a wide area in the hypochondrium and epigastrium, and the Dai-joki-To formula-pattern model featured swelling and increased resistance in the umbilical area.

With the three slight excess models, the evaluators found that: the Shigyaku-San formula-pattern model featured increased resistance to the touch in the lower hypochondrium, the epigastrium, and the abdominal rectus muscle; the Saiko-ka-ryukotsu-borei-To formula-pattern model featured increased resistance in the hypochondriac and epigastric regions; and the Keishi-bukuryo Gan formula-pattern model featured increased resistance in the lower abdomen and Oketsu tenderness.

With the intermediate strength models, the evaluators found that: the Hachimi-jio-Gan formula-pattern model featured decreased resistance in the medial lower abdomen; the Hange-shashin-To formula-pattern model featured increased resistance in the epigastric region; and the Sho-saiko-To formula-pattern model featured increased resistance in the lower hypochondrium and the epigastrium.

In the case of the slight deficiency models, the evaluators found that: the Hochu-ekki-To formula-pattern model featured increased resistance in the lower hypochondrium; the Sho-kenchu-To formula-pattern model featured increased resistance in the abdominal rectus muscle area; and that the Toki-shakuyaku-San formula-pattern model was similar to the Sho-kenchu-To formula-pattern model but also featured Oketsu tenderness in the lower abdomen.

With the two obvious deficiency models, the Ninjin-To formula-pattern model was fond to feature increased resistance in the epigastric region, and the Dai-kenchu-To formula-pattern model featured an extended area of resistance over the intestinal tract.

The evaluators determined that the producers were successful in creating the above 13 formula-pattern models, corresponding to standard Kampo formulations.

## Discussion

It does not appear to be feasible to use laboratory testing equipment to substitute for abdominal diagnoses performed by physicians; therefore, our approach is to create standard models that correspond to the patterns that physicians will encounter in clinical practice. In an earlier study, when clinical practitioners were asked to evaluate our abdominal strength models, 96.1% found them useful, which suggests that this approach has promise (Baba et al., 2018).

Reports of the usefulness of simulators in medical training exist (Ewy et al., 1987; Woolliscroft et al., 1987; Butter et al., 2010; Schubart et al., 2012). The models in the first generation of our Fukushin simulator were inferior to the present ones and gave a far from perfect reproduction of abdominal patterns, yet 149 practitioners taking our workshop on abdominal palpation said that the models were useful and had helped deepen their understanding of abdominal patterns (Yakubo et al., 2009a). With regard to the various static models included in the current Fukushin Simulator, 78.6% of educators judged them to be very useful or useful (Yakubo et al., 2009b). In general, the simulator is judged to be useful by both trainers and trainees (Yakubo et al., 2012). More recently, we conducted a practical session on abdominal diagnosis using the current version of the Fukushin simulator with medical students, and 98.4% of the students gave positive feedback (Yakubo et al., 2014b).

Arita et al. developed an education program including a general lecture on physical examination in Kampo medicine, followed by a pre-test assessment involving palpation of the simulators, a specific lecture about abdominal palpation, and finally a post-test assessment (Arita et al., 2019). Their education program using simulators for Kampo abdominal palpation can be effective and useful for beginners in Kampo medicine.

**FIGURE 11:**
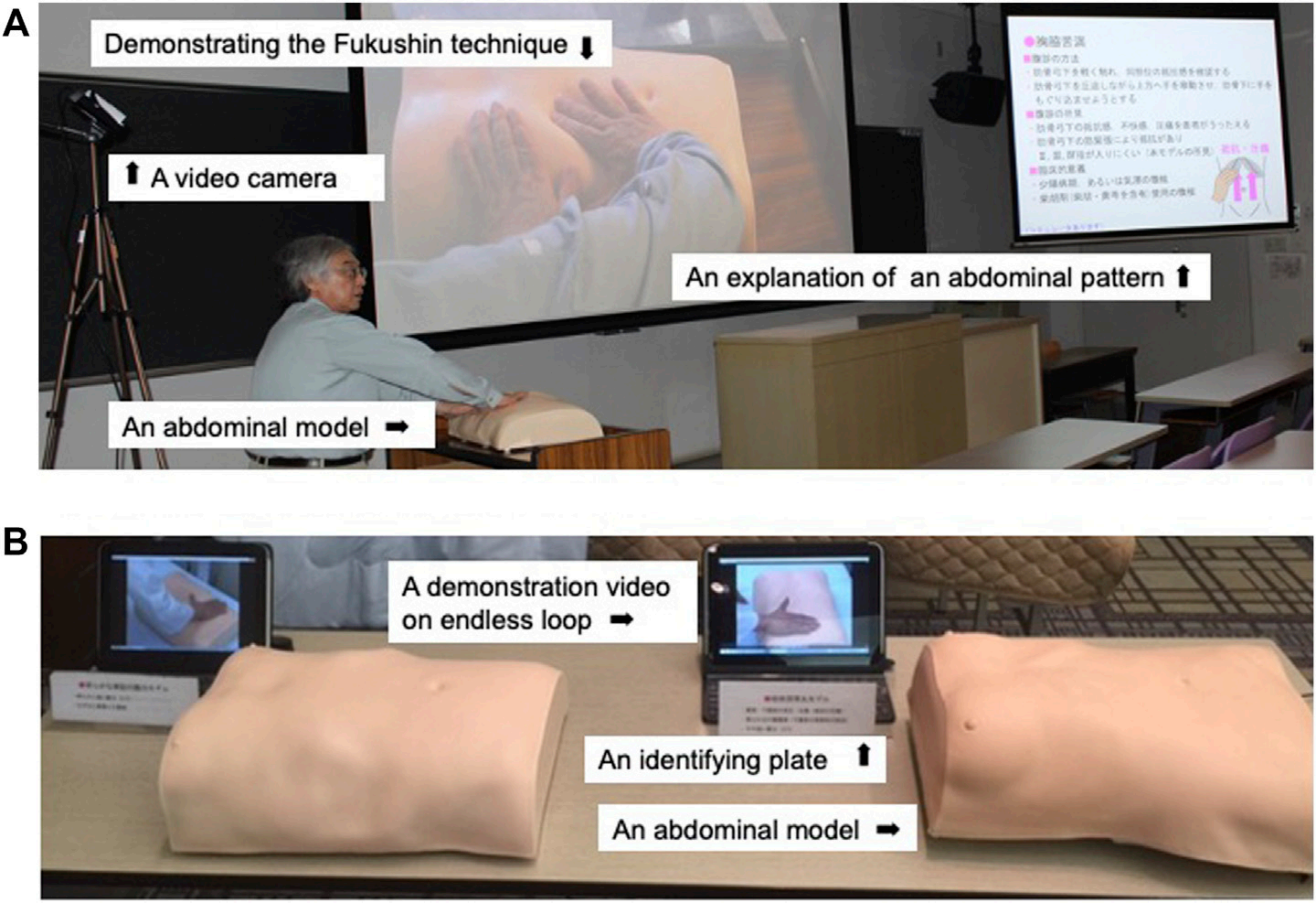
The abdominal palpation learning system in Kampo style (Abpalle Kampo). **(A)** Using an abdominal model in a lecture on Fukushin. **(B)** Placement of abdominal models and monitors.

Also recently, we have developed a teaching system for abdominal diagnosis featuring our abdominal models, called the Abdominal palpation learning system in Kampo style (Abpalle Kampo) (Yakubo et al., 2021a), which appears to have benefit in making it easier for participants to understand abdominal patterns and the corresponding herbal formulas and to learn the technique of abdominal palpation. Figure 11A demonstrates the use of an abdominal model in a lecture on Fukushin and Figure 11B shows the placement of abdominal models and monitors.

**FIGURE 12:**
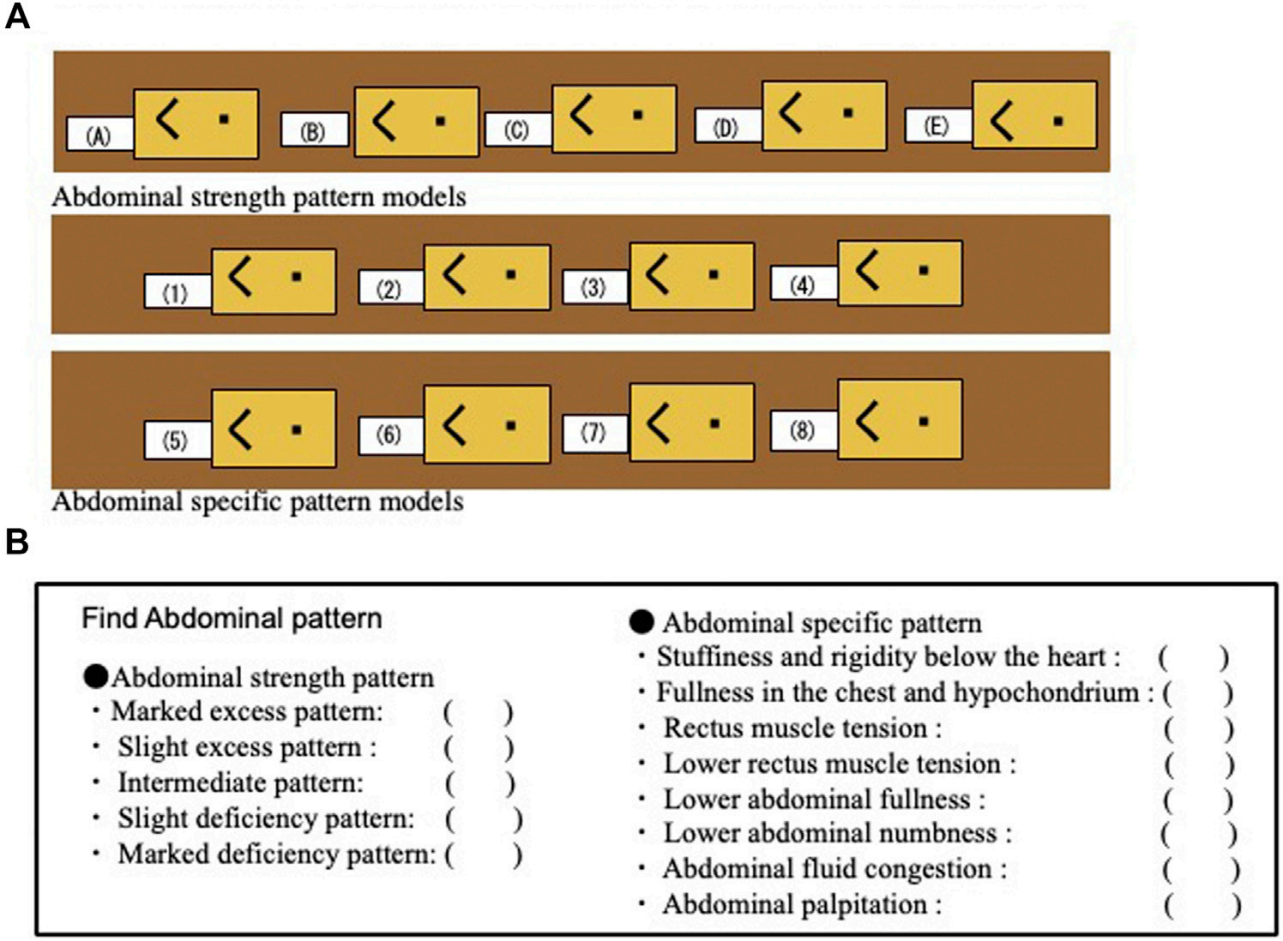
A system of Abpalle KAMPO for students. **(A)** Arrangement of abdominal strength pattern models and abdominal specific pattern models. **(B)** Student’s quiz paper.

For medical students, some of whom are reluctant to take an active part, we prepared Abdominal strength pattern models (five gradations) and eight Abdominal specific pattern models with the Fukushin simulator, labeled not with the name of the model but a number or letter and arranged at random around the room (Figure 12A) (Yakubo et al., 2021b). Students are required to go around the room and perform abdominal diagnosis on the different models, attempting to write the correct diagnosis for each one (Figure 12B). We think that it is desirable to incorporate this type of training (Abpalle KAMPO for students) as a standard part of Fukushin education for students.

For the present research, we created 13 formula-pattern models representing 13 abdominal patterns each matched to a standard Kampo formula. Using these models, it should become possible for participants to really understand the explanations of the abdominal patterns as well as the required technique. The participants can then perform diagnosis themselves on the models, which should lead to deepening their practical understanding.

Although we do not currently have case reports that list the chief complaint, history of present illness, and medical history alongside the results of abdominal diagnosis, one idea we are exploring currently is to prepare case reports featuring the results of tongue and pulse diagnosis and other available information and give them to workshop participants with an abdominal model whose name has been obscured. Participants would attempt to perform abdominal palpation and prescribe the Kampo formula that best matches the case. We can expect that this will contribute to educating practitioners with greater practical expertise. Accordingly, we believe that these 13 new models have a major contribution to make.

The World Health Organization, in its International Classification of Diseases 11th Revision, now includes mention of the standardization of traditional medical practices (Yakubo et al., 2019; World Health Organization, 2020). Abdominal diagnosis is one of the traditional medical practices mentioned, and its standardization has become an important issue. It is our hope that the models in the Fukushin simulator described in this paper will make a significant contribution to this effort.

## Conclusion

The diagnostic method known as abdominal palpation is of great importance in Kampo medicine, enabling a Kampo practitioner to determine the most suitable formulation for a patient. Based on our previous work developing a Fukushin simulator consisting of a set of abdominal models recreating important abdominal patterns, the present research describes 13 new standard female models reproducing clinically important abdominal patterns matched to Kampo formulations, which we have called formula-pattern models. These are expected to make a significant contribution to the education and standardization of abdominal palpation.

## Data Availability

The original contributions presented in the study are included in the article/Supplementary Material, further inquiries can be directed to the corresponding author.

## References

[B1] Arichi S. Akamaru S. Tani T. (1983). An Application of Kampo Abdominal Palpation to the Modern Medicine-By thermal Heart Video System (1). Igaku-to-Yakugaku 13, 667–674. (In Japanese).

[B2] Arita R. Numata T. Saito N. Takayama S. Togashi T. Kaneko S. (2019). Development of a Medical Education Program with Abdominal Palpation Simulators to Support the Understanding of Traditional Japanese (Kampo) Medicine in Beginners. Traditional Kampo Med. 6 (3), 148–155. 10.1002/tkm2.1230

[B3] Arai M. Hioki C. Kosoto H. (2017).Textbook of Traditional Japanese Medicine, Part 1: Kampo. Available at: http://kampotextbook.sakura.ne.jp/pdf/Part1_Kampo_Textbook_of_Traditional_Japanese_Medicine_en.pdf .

[B4] Baba M. Fukuda E. Yakubo S. (2018). Evaluation of Standard Abdominal Strength Pattern Models in an Abdominal Palpation Simulator and of the Standardization Project Itself. Int. Med. J. 25 (1), 1–3.

[B5] Baba M. Fukuda E. Yakubo S. (2019). Modification to an Abdominal Diagnosis Simulator to Educate Standard Abdominal Patterns of Toki-Shakuyaku-San or Keishi-Bukuryo-Gan in Kampo Medicine. Int. Med. J. 26 (1), 39–42.

[B6] Butter J. McGaghie W. C. Cohen E. R. Kaye M. Wayne D. B. (2010). Simulation-Based Mastery Learning Improves Cardiac Auscultation Skills in Medical Students. J. Gen. Intern. Med. 25, 780–785. 10.1007/s11606-010-1309-x 20339952 PMC2896602

[B7] Ewy G. A. Felner J. M. Juul D. Mayer J. W. Sajid A. W. Waugh R. A. (1987). Test of a Cardiology Patient Simulator with Students in Fourth-Year Electives. J. Med. Educ. 62 (9), 738–743. 10.1097/00001888-198709000-00005 3625738

[B8] Miyamoto K. Okita K. (2005). Reappearance and Changes of Sub-navel Hyposthenia (SNH) in Evaluation of SNH by Digital Abdominal Diagnometer (DAD). Kampo Newest Ther. 13, 185–191. (In Japanese).

[B9] Nishida Y. Narahara H. Oribe K. (2012). Anatomical Evaluation of Shofukukyuketsu by 3D Image Analysis. Kampo Med. 61, 856–859. Japanese (Summary in English). 10.3937/kampomed.61.856

[B10] Protnikoff G. A. WatanabeYashiro K. K. (2008). Kampo, from Old Wisdom Comes New Knowledge. Herbal Gram 78, 46–57.

[B11] Schubart J. R. Erdahl L. Smith J. S. Jr Purichia H. Kauffman G. L. Kass R. B. (2012). Use of Breast Simulators Compared with Standardized Patients in Teaching the Clinical Breast Examination to Medical Students. J. Surg. Educ. 69, 416–422. 10.1016/j.jsurg.2011.10.005 22483147

[B12] Shintani T. Tosa H. Yamamoto T. Imadaya A. Terasawa K. (1989). On the Relationship between X-ray Findings of Barium Enema, Abdominal Palpation Signs of Kampoh Medicine and Effective Kampoh Formulas. Kampo Med. Nihon Toyo Igaku Zasshi 39, 245–252. (Summary in English). 10.3937/kampomed.39.245

[B13] Terasawa K. (1993). Kampo, Japanese-Oriental Medicine, Insights from Clinical Cases. Tokyo: Standard McIntyre.

[B14] The Japan Society for Oriental Medicine (2005). Introduction to Kampo Japanese Traditional Medicine. Tokyo: Elsevier Japan K.K.

[B15] Tosa H. Terasawa K. Imadaya A. Mitsuma T. Matsumoto M. (1982). A Study of the Mechanism of &ldquo;INAI-TEISUI&rdquo; (Water-Imbalance Syndrome in Kampoh Medicine) -The First Report-. Nihon Toyo igaku zasshi 33, 53–58. (Summary in English). 10.3937/kampomed.33.53

[B16] Ushiroyama T. (2005). Japanese Kampo Medicine for Women: Historical Perspectives of Koho-Ha School and Current Concerns in Menopausal Medicine. Adv. Obst. Gynecol. 57, 131–149. 10.11437/sanpunosinpo.57.131

[B17] Woolliscroft J. O. Calhoun J. G. Tenhaken J. D. Judge R. D. (1987). Harvey: the Impact of a Cardiovascular Teaching Simulator on Student Skill Acquisition. Med. Teach. 9, 53–57. 10.3109/01421598709028980 3669990

[B18] World Health Organization (2020). The Supplementary Chapter 26, Traditional Medicine Conditions-Module I, International Classification of Diseases 11th Revision. (Geneva, Switzerland: WHO) Available at: https://icd.who.int/browse11/l-m/en#/http%3a%2f%2fid.who.int%2ficd%2fentity%2f718687701 .

[B19] Yakubo S. Baba M. Fukuda E. (2021a). A New Method for Training Medical Students in Abdominal Diagnosis in Kampo Style through Use of a Simulator. Int. Med. 28 (5), 539–541.

[B20] Yakubo S. Baba M. Fukuda E. (2021b). Developing an Abdominal Palpation Learning System in Kampo Style (Abpalle KAMPO) for Doctors. Int. Med. J. 28 (2), 243–245.

[B21] Yakubo S. Kinoshita Y. Ota H. (2009a). Evaluation by Clinicians Learning Kampo Medicine of a Simulator for Learning Abdominal Palpation. J. Med. Educ. Jpn. 40, 55–60. Japanese (Summary in English). 10.11307/mededjapan.40.55

[B22] Yakubo S. Kinoshita Y. Ueda Y. (2009b). Evaluation by Kampo Medical Faculty of a Simulator for Teaching Abdominal Palpation. J. Trad. Med. 26, 104–109.

[B23] Yakubo S. Ueda Y. Ishino S. (2014a). The Development of an Abdominal Palpitation Model for the Fukushin Simulator: towards Improvement and Standardization of Kampo Abdominal Diagnosis. Int. Med. J. 21 (2), 1–4.

[B24] Yakubo S. Ueda Y. Muroga K. (2014b). Students’ Impressions of an Abdominal Diagnosis Workshop Using the Fukushin Simulator. Int. Med. J. 21 (4), 358–361.

[B25] Yakubo S. Ueda Y. Ishino S. (2013). Towards the Standardization of Abdominal Strength in the Abdominal Palpation Diagnostic System of Kampo Medicine: Development of an Abdominal Strength Model in the Fukushin Simulator. Int. Med. J. 20 (6), 696–698.

[B26] Yakubo S. Ueda Y. Kinoshita Y. (2012). Making and Evaluation of a Simulator for the Teaching or Learning of Abdominal Pattern in the Japanese Kampo Style by Clinical Doctors and Educational Faculty. Int. Med. J. 19 (2), 112–114.

[B27] Yakubo S. Kinoshita Y. Aki T. Ota H. (2008). Improvement of A Simulator Production Project for Abdominal Palpation in Kampo Medical Training. Kampo Med. 59, 595–600. Japanese (Summary in English). 10.3937/kampomed.59.595

[B28] Yakubo S. Namiki T. Ito M. (2019). Chapter 26 Traditional Medicine Included in ICD-11 Has Been Released, till Now and from Now on!. Nihon Toyo igaku zasshi 70 (2), 167–174. (Summary in Japanese). 10.3937/kampomed.70.167

[B29] Yakubo S. Ueda Y. Muroga K. Tanekura N. Okudaira T. Sasanuma T. (2015). Modifications to an Abdominal Diagnosis Simulator to Reproduce Patterns Characterized by Local Variations in Resistance to Pressure. Traditional Kampo Med. 2 (2), 31–34. 10.1002/tkm2.1015

[B30] Yasaka T. (1994). Analitical Use of Ultrasonography in the Signs of "Saikafujin". Kampo Med. Nihon Toyo Igaku Zasshi 45, 331–337. Japanese (Summary in English). 10.3937/kampomed.45.331

